# A review of deep learning applications in human genomics using next-generation sequencing data

**DOI:** 10.1186/s40246-022-00396-x

**Published:** 2022-07-25

**Authors:** Wardah S. Alharbi, Mamoon Rashid

**Affiliations:** Department of AI and Bioinformatics, King Abdullah International Medical Research Center (KAIMRC), King Saud Bin Abdulaziz University for Health Sciences (KSAU-HS), King Abdulaziz Medical City, Ministry of National Guard Health Affairs, P.O. Box 22490, Riyadh, 11426 Saudi Arabia

**Keywords:** Human genomics, Deep learning applications, Disease variants, Gene expression, Epigenomics, Pharmacogenomics, Variant calling, NGS

## Abstract

Genomics is advancing towards data-driven science. Through the advent of high-throughput data generating technologies in human genomics, we are overwhelmed with the heap of genomic data. To extract knowledge and pattern out of this genomic data, artificial intelligence especially deep learning methods has been instrumental. In the current review, we address development and application of deep learning methods/models in different subarea of human genomics. We assessed over- and under-charted area of genomics by deep learning techniques. Deep learning algorithms underlying the genomic tools have been discussed briefly in later part of this review. Finally, we discussed briefly about the late application of deep learning tools in genomic. Conclusively, this review is timely for biotechnology or genomic scientists in order to guide them why, when and how to use deep learning methods to analyse human genomic data.

## Introduction

Understanding the genomes of diverse species, specifically, the examination of more than 3 billion base-pairs of Homo sapiens DNA, is a crucial aim of genomic studies. Genomics takes a comprehensive view that implicates all the genes within an organism, including protein-coding genes, RNA genes, *cis-* and *trans-*elements, etc. It is a data-driven science involving the high-throughput technological development of next-generation sequencing (NGS) that generates the entire DNA data of an organism. These techniques include whole genome sequencing (WGS), whole exome sequencing (WES), transcriptomic and proteomic profiling [1–5]. With the recent rapid accumulation of these omics data, increased attention has been paid to bioinformatics and machine learning (ML) tools with established superior performance in several genomics implementations [6]. These implementations involve finding a genotype–phenotype correlation, biomarker identification and gene function prediction, as well as mapping the biomedically active genomic regions, for example, transcriptional enhancers [7–10].

Machine learning (ML) has been deliberated as a core technology in artificial intelligence (AI), which enables the use of algorithms and makes critical predictions based on data learning and not simply following instructions. It has broad technology applications; however, standard ML methods are too narrow to deal with complex, natural, highly dimensional raw data, such as those of genomics. Alternatively, the deep learning (DL) approach is a promising and exciting field currently employed in genomics. It is an ML derivative that extracts features by applying neural networks (NN) automatically [11–14]. Deep learning has been effectively applied in fields such as image recognition, audio classification, natural language processing, online web tools, chatbots and robotics. In this regard, the utilisation of DL as a genomic methodology is totally apt to analyse a large amount of data. While it is still in its infant stages, DL in genomics holds the promise of updating arenas such as clinical genetics and functional genomics [15]. Undoubtedly, DL algorithms have dominated computational modelling approaches in which they are currently regularly expanded to report a variety of genomics questions ranging from understanding the effects of mutations on protein–RNA binding [16], prioritising variants and genes, diagnosing patients with rare genetic disorders [17], predicting gene expression levels from histone modification data [18] and to identifying trait-associated single-nucleotide polymorphisms (SNPs) [19].

**Fig. 1:**
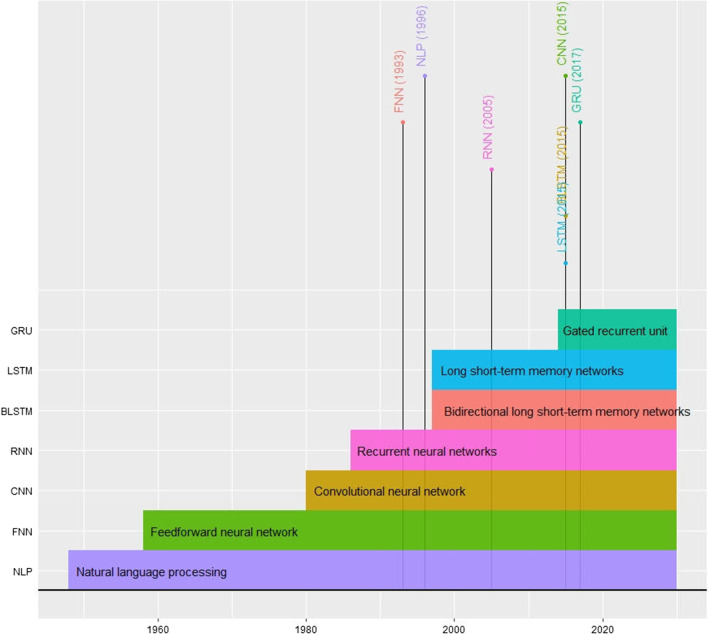
Timeline of implementing deep learning algorithms in genomics. This timeline plot demonstrated the delay of implementing DL tools in genomics; for example, both (LSTM) and (BLSTM) algorithms have been invented in 1997 and the first genomic application was implemented in 2015. Similar observations are for the rest of the deep learning algorithms (Table 6)

Although the first concept of the DL theory originated in the 1980s was based on the perceptron model and neuron concept [20], within the last decade, DL algorithms have become a state-of-the-art predictive technology for big data [21–23]. The initial efficient implementation of DL prediction models in genomics was in the 2000s (Fig. 1) [24]. The difficulty associated with the requirement of DL models to train an enormous amount of training datasets and the need for powerful computing resources limited their applications until the introduction of modern hardware, such as the high-efficiency graphical processing units (GPUs) with equivalent structures. Now, the architectures of DL models (also known as DNNs) are implemented in diverse areas, as mentioned earlier. Classical neural networks consist of only two to three hidden layers; however, DL networks extend this up to 200 layers. Thus, the word “deep” reflects the number of layers that the information passes through. However, DL requires superior hardware and substantial parallelism to be applicable [25]. Due to overwhelmed hardware limitations and demanding resources, several DL packages and resources were introduced to facilitate DL model implementation (discussed in section deep learning resources for genomics).

The evolution of software, hardware (GPUs) and big data in genomics has facilitated the development of deep learning-based prediction models for the prediction of functional elements in genomes. These genetic variants from NGS data predict splice sites in genomic DNA, predict the transcription factor binding sites (TFBSs) via classification tasks, classify the pathogenicity of missense mutations and predict drug response and synergy [26–31]. An example of a technological evolution that has enhanced DL implementation is cloud platforms, which provide GPU resources as a DL solution. GPUs can considerably escalate the training speed as the neural network training style can be more adaptable in certain model architecture situations, thus permitting fast mathematical processes through the use of larger processing unit numbers and high-memory capacities. Primary examples of cloud computing platforms include Amazon Web Services, Google Compute Engine and Microsoft Azure. However, these elucidations still require users to implement model codes [32].

For all ML models, the evaluation metrics are essential in understanding the model performance. Basically, these metrics are crucial to be considered in case of genomic datasets which generate naturally a highly imbalanced classes that makes them demanding to be applied by ML and DL models. A sufficient number of solutions usually applied in this case such as transfer learning [33] and Matthews correlation coefficient (MCC) [34]. In common sense, every ML task can be divided into a regression task (e.g. predicting certain outcomes/effects of a disease) or a classification task (e.g. predicting the presence/absence of a disease); additionally, multiple measurement metrics are obtained from those tasks. Generally, some, but not all, performance metrics used in ML regression-based methods include: mean absolute error (MAE), mean squared error (MSE), root-mean-squared error (RMSE) and coefficient of determination (R^2^). In contrast, the performance metrics in ML classification-based methods include: accuracy, confusion matrix, area under the curve (AUC) or/and area under receiver operating characteristics (AUROC) and F1-score. The classification tasks are most commonly applied to problems in research areas in genomics and for comparing different models’ performance. For example, AUC is the most widely used metric for evaluating the model performance ranging from [0, 1]. It measures the true-positive rate (TPR) or sensitivity, true-negative rate (TNR) or specificity and the false-positive rate (FPR). Additionally, the F1-score is used to test the model accuracy in highly imbalanced dataset and is the harmonic mean between the precision and recall (also ranging from [0, 1]). For both AUC and F1-score, a greater value reflects better model performance. Also, the confusion matrix describes the complete model performance by measuring the model accuracy to calculate true-positive values plus true-negative values and dividing the sum over the total number of samples [35, 36]. For a greater understanding of the ML evaluation metrics—purpose, calculation, etc.—recommended papers include Handelman et al. (2019) and England and Cheng (2019).

This article reviews deep learning tools/methods based on their current applications in human genomics. We began by collecting recent (i.e. published in 2015–2020) DL tools in five main genomics areas: variant calling and annotation, disease variants, gene expression and regulation, epigenomics and pharmacogenomics. Then, we briefly discussed DL genomics-based algorithms and their application strategies and data structure. Finally, we mentioned DL-based practical resources to facilitate DL adoption that would be extremely beneficial mostly to biomedical researchers and scientists working in human genomics. For further information on the field of DL applications in genomics, we recommend: [37–39].

## Deep learning tools/software/pipelines in genomics

**Fig. 2:**
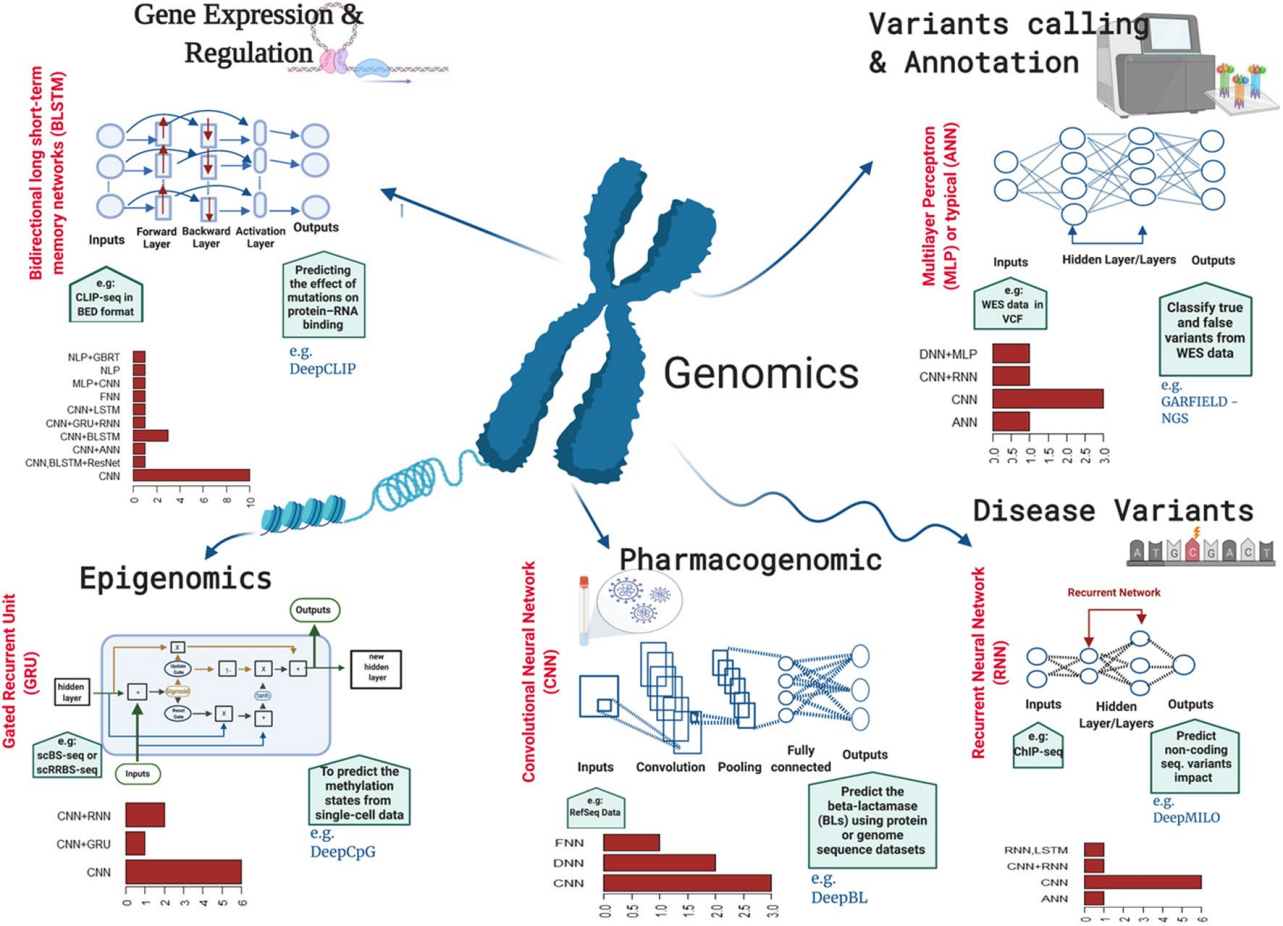
Deep learning applications in genomics. This figure represents the application of deep learning tools in five major subareas of genomics. One example deep learning tool and underlying network architecture has been shown for each of the genomic subareas, and its input data type and the predictive output were mentioned briefly. Each bar plot depicts the frequency of most used deep learning algorithms underlying deep learning tools in that subarea of genomics (Tables 1, 2, 3, 4, 5)

Multiple genomic disciplines (e.g. variant calling and annotation, disease variant prediction, gene expression and regulation, epigenomics and pharmacogenomics) take advantage of generating high-throughput data and utilising the power of deep learning algorithms for sophisticated predictions (Fig. 2). The modern evolution of DNA/RNA sequencing technologies and machine learning algorithms especially deep learning opens a new chapter of research capable of transforming big biological data into new knowledge or novel findings in all subareas of genomics. The following sections will discuss the latest software/tools/pipelines developed using deep learning algorithms in various genomics areas.

### Variant calling and annotation

This first section discusses the applications of the latest DL algorithms in variant calling and annotation. We provided a short list of tools/algorithms for variant calling and annotation with their source code links, if available (Table 1), to facilitate the selection of the most suitable DL tool for a particular data type.

**Table 1:** Genomic tools/algorithm based on deep learning architecture for variant calling and annotations

Tools	DL model	Application	Input/Output	Website Code Source	References
Clairvoyante	CNN	To predict variant type, zygosity, alternative allele and Indel length	BAM/VCF	https://github.com/aquaskyline/Clairvoyante	[145]
DeepVariant	CNN	To call genetic variants from next-generation DNA sequencing data	BAM,CRAM/VCF	https://github.com/google/deepvariant	[30]
GARFIELD-NGS	DNN + MLP	To classify true and false variants from WES data	VCF/VCF	https://github.com/gedoardo83/GARFIELD-NGS	[146]
Intelli-NGS	ANN	To define good and bad variant calls from Ion Torrent sequencer data	VCF/xlsx	https://github.com/aditya-88/intelli-ngs	[147]
DAVI (Deep Alignment and Variant Identification)	CNN + RNN	To identify variants in NGS reads	FASTQ/VCF	N/A	[116]
DeepSV	CNN	To call genomic deletions by visualising sequence reads	BAM/VCF	https://github.com/CSuperlei/DeepSV	[52]

NGS, including whole genome or exome, sets the stage for early developments in personalised medicine, along with its known implications in Mendelian disease research. With the advent of massively parallel, high-throughput sequencing, sequencing thousands of human genomes to identify genetic variations has become a routine practice in genomics, including cancer research. Sophisticated bioinformatics and statistical frameworks are available for variant calling.

The weakness of high-throughput sequencing procedures is represented by significantly high technical and bioinformatics error rates [40–42]. Numerous computational problems have originated due to the enormous amounts of medium or low coverage genome sequences, short read fragments and genetic variations among individuals [43]. Such weaknesses make the NGS data dependent on bioinformatics tools for data interpretation. For instance, several variant calling tools are broadly used in clinical genomic variant analyses, such as genome analysis toolkit (GATK) [44], SAMtools [45], Freebayes [46] and Torrent Variant Caller (TVC; [47]). However, despite the availability of whole genome sequencing, some actual variants are yet to be discovered [48].

Contemporary deep learning tools have been proposed in the field of next-generation sequencing to overcome the limitations of conventional interpretation pipelines. For example, Kumaran et al*.* demonstrated that combining DeepVariant, a deep learning-based variant caller, with conventional variant callers (e.g. SAMtools and GATK) improved the accuracy scores of single-nucleotide variants and Indel detections [49]. Implementing deep learning algorithms in DNA sequencing data interpretation is in its infancy, as seen with the recent pioneering example, DeepVariant, developed by Google. DeepVariant relies on the graphical dissimilarities in input images to perform the classification task for genetic variant calling from NGS short reads. It treats the mapped sequencing datasets as images and converts the variant calls into image classification tasks [30]. However, this model does not provide details about the variant information, for example, the exact alternative allele and type of variant. As such, it is classified as an incomplete variant caller model [50].

Later, several DL models for variant calling and annotation were introduced. For instance, Cai et al. (2019) introduced DeepSV, a genetic variant caller that aims to predict long genomic deletions (> 50 bp) extracted from sequencing read images but not other types of structural variants, such as long insertions or inversions. It processes the BAM format or VCF files as inputs and outputs the results in the VCF form. In terms of evaluating DeepSV, it was compared with another eight deletion calling tools and one machine learning-based tool called Concod [51]. The results reveal that although Concod has shorter training times in the case of fewer trained samples, DeepSV shows a higher accuracy score and fewer training losses using the same dataset [52]. Another genomic variant filtering tool, GARFIELD-NGS, can be applied directly to the variant caller outputs. It relies on an MLP algorithm to investigate the true and false variants in exome sequencing datasets generated from the Ion Torrent and Illumina platforms. It represents a robust performance at low coverage data (up to 30X) by handling standard VCF file, resulting in another VCF file. Ravasio et al. (2018) observed that the GARFIELD-NGS model recorded a significant reduction in the false candidate variants after applying a canonical pipeline for the variant prioritisation of disease-related data [53].

The Clairvoyante model was introduced to predict variant type (SNP or Indel), zygosity, allele alternative and Indel length. Thus, it overcomes the DeepVariant model’s drawback of lacking the full variant details, including the precise alternative allele and variant type. The Clairvoyante model was specifically designed to utilise long-read sequencing data generated from SMS technologies (e.g. PacBio and ONT), although it is commonly applicable for short read datasets as well [50]. Another variant caller and annotation model, Intelli-NGS, was introduced by Singh and Bhatia (2019). One variant calling was based on artificial neural network (ANN), which utilises the data generated from the Ion Torrent platform to identify true and false effectively. Intelli-NGS takes any number of VCF files as batch inputs and processes them in order. The processed data results in an excel sheet related to each VCF file containing the HGVS codes of all variants [54]. All in all, several studies confirmed the capabilities of deep learning in genetic variant calling and annotation from sequencing data.

### Disease variants

Deep learning-based models for the prediction of pathogenic variants, their application and input/output formats with source codes (if available) are listed in Table 2.

**Table 2:** Genomic tools/algorithm based on deep learning architecture for disease variants

Tools	DL model	Application	Input/Output	Website Code Source	References
DeepPVP (PhenomeNet Variant Predictor)	ANN	to identify the variants in both whole exome or whole genome sequence data	VCF / VCF	https://github.com/bio-ontology-research-group/phenomenet-vp	[61]
ExPecto	CNN	Accurately predict tissue-specific transcriptional effects of mutations/functional SNPs	VCF/ CSV	https://github.com/FunctionLab/ExPecto	[138]
PEDIA (Prioritisation of exome data by image analysis)	CNN	To prioritise variants and genes for diagnosis of patients with rare genetic disorders	VCF / CSV	https://github.com/PEDIA-Charite/PEDIA-workflow	[148]
DeepMILO (Deep learning for Modeling Insulator Loops)	CNN + RNN	to predict the impact of non-coding sequence variants on 3D chromatin structure	FASTA / TSV	https://github.com/khuranalab/DeepMILO	[119]
DeepWAS	CNN	To identify disease or trait-associated SNPs	TSV / TSV	https://github.com/cellmapslab/DeepWAS	[19]
PrimateAI	CNN	To classify the pathogenicity of missense mutations	CSV / CSV + txt	https://github.com/Illumina/PrimateAI	[27]
DeepGestalt	CNN	To Identifying facial phenotypes of genetic disorders	Image / txt	Is available through the Face2Gene application, http://face2gene.com	[149]
DeepMiRGene	RNN, LSTM	To predict miRNA precursor	FASTA / Cross-Validation (CV)-Splits file	https://github.com/eleventh83/deepMiRGene	[150]
Basset	CNN	To predict the causative SNP with sets of related variants	BED, FASTA/ VCF	https://github.com/davek44/Basset	[151]

Considering extra data from patient relatives or relevant cohorts, medical geneticists frequently prioritise and filter the observed genetic variants after variant calling and annotation (Müller et al. [55]). Variant prioritisation is a method of determining the most likely pathogenic variant within genetic screening that damages gene function and underlying the disease phenotype [56]. Variant prioritisation involves variant annotation to discover clinically insignificant variants, such as synonymous, deep-intronic variants and benign polymorphisms. Subsequently, the remaining variants, such as known variants or variants of unknown clinical significance (VUSs), become attainable [57]. Furthermore, complications in interpreting rare genetic variants in individuals, for example, and understanding their impacts on disorder risk influence the clinical capability of diagnostic sequencing. For example, the numerous and infrequent VUSs in rare genetic diseases represent a challenging obstacle in sequencing implementation for personalised medicine and healthy population assessment (Sundaram et al., 2018). Although statistical methods, such as GWAS, have had huge success in combining genetic variants to disorders, they still require heavy sampling to distinguish rare genetic variants and cannot deliver information about de novo variants (Fu et al., 2014). Thus, current annotation approaches, such as PolyPhen [58], SIFT [59] and GERP [60], represent beneficial methods for prioritising the causative variants, despite facing some drawbacks. For such problems, DL-based models have been implemented to enable a powerful method for exploiting the deep neural network (DNN) architecture to prioritise variants, for instance, the Basset model, a variant annotator, that relies on a CNN algorithm and is designed to predict the causative SNP exploiting DNase I hypersensitivity sequencing data as an input (Kelley, Snoek and Rinn, 2016).

The clinical and molecular validations cannot be replaced by in silico prediction models; however, in a sense, they can contribute to decrease waiting times for results and can prioritise variants for further functional analysis. These predictable models are mainly suitable when several poorly understood candidate variants convey certain phenotypes [27]. Medical genetics has been significantly transformed following the proposition of NGS technology, particularly with WGS because of its power to interpret genomic variations in both coding and non-coding fragments within the entire human genome. Recently, several ML-based methods have offered to prioritise non-coding variants; still, the recognition of disease-associated variants in complex traits, such as cancers, is challenging. Plus, the majority of positive variants associated with a certain phenotype is required to predict general and precise novel correlations (Schubach et al., 2017). Lately, several DL approaches have been proposed to overcome these challenges. For example, the DeepWAS model relies on a CNN algorithm that allows regulatory impact prediction of each variant on numerous cell-type-specific chromatin features. The key result of the DeepWAS model is the direct determination of the disease-associated SNPs with a common effect on a certain chromatin trait in the related tissue. The DeepWAS model demonstrated the ability to detect the disease-relevant, transcriptionally active genomic position after combining the expression and methylation quantitative-trait loci data (eQTL and meQTL, respectively) of various resources and tissues [19]. Nevertheless, several deep learning algorithms have been described as discovering novel genes. For this reason, deep learning approaches are particularly suited for variant investigation for genes not yet related to specific disease phenotypes [61, 62].

### Gene expression and regulation

In this section, we focused on the most efficient deep learning-based tools in the area of gene expression and regulation in the genome. We listed several models applying various deep learning algorithms and summarised the information and source codes mostly in splicing and gene expression applications, if available (Table 3).

**Table 3:** Genomic tools/algorithm based on deep learning architecture for gene expression regulation

Tools	DL model	Application	Input/Output	Website Code Source	References
DanQ	CNN + BLSTM	To predict DNA function directly from sequence data	.mat /.mat	https://github.com/uci-cbcl/DanQ	[152]
SPEID	CNN + LSTM	For enhancer–promoter interaction (EPI) prediction	.mat /.mat	https://github.com/ma-compbio/SPEID	[153]
EP2vec	NLP + GBRT	To predict enhancer–promoter interactions (EPIs)	CSV / CSV	https://github.com/wanwenzeng/ep2vec	[154]
D-GEX (deep learning for gene expression)	FNN	To understand the expression of target genes from the expression of landmark genes	.cel, txt, BAM / txt	https://github.com/uci-cbcl/D-GEX	[155]
DeepExpression	CNN	To predict gene expression using promoter sequences and enhancer–promoter interactions	.txt /.txt	https://github.com/wanwenzeng/DeepExpression	[156]
DeepGSR	CNN + ANN	To recognise various types of genomic signals and regions (GSRs) in genomic DNA (e.g. splice sites and stop codon)	FASTA /.txt	https://zenodo.org/record/1117159#.Xp4B4y2B1p8	[157]
SpliceAI	CNN	To identify splice function from pre-mRNA sequencing	VCF / VCF	https://github.com/Illumina/SpliceAI	[71]
SpliceRover	CNN	For splice site prediction	FASTA /.txt	N/A	[158]
Splice2Deep	CNN	For splice site prediction in Genomic DNA	FASTA /.txt	https://github.com/SomayahAlbaradei/Splice_Deep	[29]
DeepBind	CNN	To characterise DNA- and RNA-binding protein specificity	FASTA /.txt	https://github.com/MedChaabane/DeepBind-with-PyTorch	[111]
Gene2vec	NLP	To produce a representation of genes distribution and predict gene–gene interaction	.txt /.txt	https://github.com/jingcheng-du/Gene2vec	[130]
MPRA-DragoNN	CNN	To ﻿predict and analyse the regulatory DNA sequences and non-coding genetic variants	N/A	https://github.com/kundajelab/MPRA-DragoNN	[77]
BiRen	CNN + GRU + RNN	For enhancers predictions	BED, BigWig /CSV	https://github.com/wenjiegroup/BiRen	[159]
APARENT (APA REgression NeT)	CNN	To predict and engineer the human 3' UTR Alternative Polyadenylation (APA) and annotate pathogenetic variants	FASTA / CSV	https://github.com/johli/aparent	[72]
LaBranchoR (LSTM Branchpoint Retriever)	BLSTM	To predict the location of RNA splicing branchpoint	FASTA / FASTA	https://github.com/jpaggi/labranchor	[160]
COSSMO	CNN, BLSTM + ResNet	To predict the splice site sequencing and splice factors	TSV, CSV /CSV	http://cossmo.genes.toronto.edu/	[79]
Xpresso	CNN	To predict gene expression levels from genomic sequence	FASTA /.txt	https://github.com/vagarwal87/Xpresso	[73]
DeepLoc	CNN + BLSTM	To predict subcellular localisation of protein from sequencing data	FASTA/ prediction score	https://github.com/JJAlmagro/subcellular_localization	[161]
SPOT-RNA	CNN	To predict RNA Secondary Structure	FASTA /.bpseq,.ct, and.prob	https://github.com/jaswindersingh2/SPOT-RNA/	[162]
DeepCLIP	CNN + BLSTM	For predicting the effect of mutations on protein–RNA binding	FASTA /.txt	https://github.com/deepclip/deepclip	[163]
DECRES (DEep learning for identifying Cis-Regulatory ElementS)	MLP + CNN	To predict active enhancers and promoters across the human genome	FASTA /.txt	https://github.com/yifeng-li/DECRES	[74]
DeepChrome	CNN	For prediction of gene expression levels from histone modification data	Bam / TSV	https://github.com/QData/DeepChrome	[164]
DARTS	DNN + BHT	Deep learning augmented RNA-seq analysis of transcript splicing	.txt	https://github.com/Xinglab/DARTS	

Gene expression involves the initial transcriptional regulators (e.g. pre-mRNA splicing, transcription and polyadenylation) to functional protein production [63]. The high-throughput screening technologies that test thousands of synthetic sequences have provided rich knowledge concerning the quantitative regulation of gene expression, although with some limitations. The main limitation is that huge biological sequence regions cannot be explored using experimental or computational techniques [64]. Although recent NGS technology has provided great knowledge in the gene-regulation field, the majority of natural mRNA screening approaches still utilise chromatin accessibility, ChIP-seq and DNase-seq information; they focus on studying promoter regions. Therefore, a robust method is required to understand the relationship between various regions of gene regulatory structures and their networks expression connection [65]. Likewise, the current technology in RNA sequencing has empowered the direct sequencing of single cells, identified as single-cell RNA sequencing (scRNA-seq), that permits querying biological systems at unique intention. For example, the data of scRNA-seq produce valuable information into cellular heterogeneity that could expand the interpretation of human diseases and biology [66, 67]. Its major applications of scRNA-seq data understanding involved in detecting the type and state of the cells [68, 69]. However, the two main computational questions include how to cluster the data and how to retrieve them [70].

Deep learning has empowered essential progress for constructing predictive methods linking regulatory sequence elements to the molecular phenotypes [71–74]. Just recently, Gundogdu and his colleagues (2022) demonstrate an excellent classification model based on deep neural networks (DNNs). It constricted numerous types of previous biological information on functional networks between genes to understand a biological significant illustration of the scRNA-seq data [70]. Moreover, Li et al. (2020) present a DESC an unsupervised deep learning algorithm implemented based on python, which understands iteratively representation of cluster-specific gene expression and the scRNA-seq analysis cluster tasks [75]. Further, deep learning model has also been applied for single-cell sequencing data. Its deep neural network (DNN) model designed to measure the immune infiltration in both colorectal and breast cancers bulk scRNA-seq data. This approach permits quantifying a particular type of immune cells such as CD8 + and CD4Tmem plus the general population of lymphocytes together with Stromal content and B cells [76].

Recently, Jaganathan et al*.* (2019) constructed SpliceAI, a deep residual neural network that predicts splice function using only pre-mRNA transcript sequencing as inputs. An architecture contained a 32-dilated convolutional layer employed to identify sequence determinates crossing enormous genomic gaps since there are tens of thousands of nucleotides separated splice-donors and splice-acceptors [71].

Many experimental datasets, such as the ChIP-seq and DNase-seq assays, do not measure the effects on gene expression directly; however, they are an ideal complement to deep neural network methods. For instance, Movva et al. (2019) introduced the MPRA-DragoNN model, based on CNN architecture for prediction and analysis of the transcription regulatory activity of non-coding DNA sequencing data measured from (MPRAs) data. Approximately 16 K distinct regulatory regions in K562 and HepG2 cell lines of 295 bp *cis*-regulatory elements cloned upstream of either minimal-promoter or strong-promoter used in the Sharpr-MPRA evaluation [77]. A very contemporary DL model, introduced by Agarwal and Shendure, named the Xpresso model, a deep convolutional neural network (CNN), conjointly models the promoter sequence and its related mRNA stability features to predict the gene expression levels of mRNA. Interestingly, Xpresso models are simple to train at several arbitrary cell types, even when they lack experimental information, such as ChIP and DNase [73]. Zhang Z. et al. (2019) developed a deep learning-based model called DARTS; deep learning augmented RNA-seq analysis of transcript splicing, that use a wide-ranging RNA-seq resources of a various alternative splicing. It consists of two main modules: deep neural network (DNN) and Bayesian hypothesis testing (BHT) [78]. More DL-based models (specifically, four different CNN architectures) designed by Bretschneider et al. (2018), named the competitive splice site model (COSSMO), which adapts to various quantities of alternative splice sites and precisely estimates them via genome-wide cross-validation. The frameworks consist of convolutional layers, communication layers, long short-term memory (LSTM) and residual networks, correspondingly, to discover related motifs from DNA sequences. In every putative splice site, the used model inputs are DNA and RNA sequences with 80 nucleotide-wide windows around the alternative splice sites and opposite constitutive splice sites together with the intron length. The outputs of the model are predictions of percent selected index (PSI) distribution of every putative splice-site. All of COSSMO model’s performance exceeds MaxEntScan; however, there were large performance variances among the four frameworks, in which recurrent LSTM reached the best accuracy over the communication networks, which did not consider the splice-site ordering [79]. However, to learn the automated relationships among heterogeneous datasets in imperfect biological situations, deep learning models offer unprecedented opportunities.

### Epigenomics

This section discusses some epigenomics challenges and summarises up-to-date deep learning models in epigenomics, their implementation, data types and source code (Table 4). Modifications in phenotypes that are not based on genotype modifications are referred to as epigenetics. It is defined as the study of heritable modifications in gene expressions which does not include DNA sequence modifications [80]. Epigenomic mechanisms, including DNA methylation, histone modifications and non-coding RNAs, are considered fundamental in understanding disease developments and finding new treatment targets. Although in clinical implementations, epigenetics has yet to be completely employed. Recently, complications initiated in developing data interpretation tools to advances in next-generation sequencing and microarray technology to produce epigenetic data. The insufficiency of suitable and efficient computational approaches has led current research to focus on a specific epigenetic mark separately, although several mark interactions and genotypes occurred in vivo [81]. Several previous studies have disclosed the fundamental applications of deep learning models in epigenomics. They reached unlimited success in predicting 3D chromatin interactions, methylation status from single-cell datasets and histone modification sites based on DNase-Seq data [62, 82–84].

**Table 4:** Genomic tools/algorithm based on deep learning architecture for epigenomics

Tools	DL model	Application	Input/Output	Website Code Source	References
DeepSEA	CNN	To predict multiple chromatin effects of DNA sequence alterations	N/A	https://github.com/Team-Neptune/DeepSea	[165]
FactorNet	CNN + RNN	For predict the cell-type specific transcriptional binding factors (TF)	BED / BED, gzipped bedgraph file	https://github.com/uci-cbcl/FactorNet	[120]
DeMo (Deep Motif Dashboard)	CNN + RNN	For transcription factor binding site perdition (TFBS) by classification task	FASTA / txt	https://github.com/const-ae/Neural_Network_DNA_Demo	[166]
DeepCpG	CNN + GRU	To predict the methylation states from single-cell data	TSV / TSV	https://github.com/cangermueller/deepcpg	[83]
DeepHistone	CNN	To accurately predict histone modification sites based on sequences and DNase-Seq (experimental) data	txt, CSV / CSV	https://github.com/ucrbioinfo/DeepHistone	[84]
DeepTACT	CNN	To predict 3D chromatin interactions	CSV / CSV	https://github.com/liwenran/DeepTACT	[167]
Basenji	CNN	To predict cell-type-specific epigenetic and transcriptional profiles in large mammalian genomes	FASTA / VCF	https://github.com/calico/basenji	[114]
Deopen	CNN	To predict the chromatin accessibility from DNA sequence/ Downstream analysis also included QTL analysis	BED, hkl /hkl	https://github.com/kimmo1019/Deopen	[31]
DeepFIGV (Deep Functional Interpretation of Genetic Variants)	CNN	To predicts impact on chromatin accessibility and histone modification	FASTA / TSV	http://deepfigv.mssm.edu	[62]

Liu et al. (2018) introduced a hybrid deep CNN model, Deopen, which was applied to predict chromatin accessibility within a whole genome from learned regulatory DNA sequence codes. In order to analytically evaluate Deopen’s function in capturing the accessibility codes of a genome, a series of experiments were conducted from the perspective of binary classification [31]. As an example of Deopen applications, in the androgen-sensitive human prostate adenocarcinoma cell lines (LN-CaP), the EGR1 recovered by the Deopen model is assumed to play a critical role as a treatment target in gene therapy for prostate cancer [31, 85]. Recently, Yin et al. (2019) proposed the DeepHistone framework, a CNN-based algorithm to predict the histone modifications to various site-specific markers. For precise predictions, this model combines DNA sequence data with chromatin accessibility information. It has revealed the capability to discriminate functional SNPs from their adjacent genetic variants, thus having the possibility to be utilised for investigating functional impacts of putative disorder-related variants [84]. Hence, efficient deep learning models are necessary for genome research to elucidate the epigenomic modifications’ impact on the downstream outputs.

### Pharmacogenomics

We listed the most deliberated deep learning pharmacogenomics models, their common purposes, input/output formats and the source of code (Table 5). Although there has been a great interest in deep learning approaches in the last few years, until very recently, deep learning tools have been rarely employed for pharmacogenomics problems, such as to predict drug response [86]. Knowledge concerning the association between genetic variants in enormous gene clusters up to whole genomes and the impacts of varying drugs is called pharmacogenomics [87]. A key challenge in modern therapeutic methods is understanding the underlying mechanisms of variability. Sometimes the medication response distribution through a certain population is evidently bimodal, proposing a dominant function for one variable, which is usually genetic. Nonetheless, an understanding of the underlying mechanisms of pharmacokinetics or pharmacodynamics could be utilised to detect candidate genes, wherein the function of those gene variants could explicate various drug reactions (88). The clinical experiments generate various errors during the investigation of drug combination efficiency, which is time- and cost-intensive. Besides, it could expose the patient to excessive risky therapy [89, 90]. In order to identify alternative drug synergy strategies without harming patients, high-throughput screening (HTS) using several concentrations of a couple of drugs employed to a cancer cell line is utilised [91]. Utilising existing HTS synergy datasets allowed the use of accurate computational models to investigate an enormous synergistic space. Such reliable models would provide direction for both in vitro and in vivo studies, and they are great steps towards personalised medicine, for instance, prediction approaches of anticancer synergic, systems biology [92], kinetic methods [93] and in silico-based models of gene expression screening after single-drug and dose-reaction treatments [94]. Nonetheless, these approaches are limited to particular targets, pathways or certain cell lines and sometimes need a particular omics dataset of treated cell lines with specific compounds [95].

**Table 5:** Genomic tools/algorithm based on deep learning architecture for pharmacogenomics

Tools	Function	DL model	Application	Input/Output	Website Code Source	References
DeepDR	Drug Repositioning	DNN	To translate pharmacogenomics features identified from in vitro drug screening to predict the response of tumours	txt / txt	https://github.com/ChengF-Lab/deepDR	[97]
DNN-DTI (Drug–target interaction prediction)	Database	DNN	To predict drug-target interaction	txt / txt	https://github.com/JohnnyY8/DNN-DTI	[168]
DeepBL	Antibiotic Resistance	CNN	To predict the beta-lactamase (BLs) using protein or genome sequence datasets	FASTA / CSV	http://deepbl.erc.monash.edu.au	[98]
DeepDrug3D	Binding Site for drugs	CNN	To characterise and classify the protein 3D binding pockets	pdb / txt	https://github.com/pulimeng/DeepDrug3D	[115]
DrugCell	Drug response and synergy for cancer cells	CNN	To predict drug response and synergy	txt / txt	https://github.com/idekerlab/DrugCell	[26]
DeepSynergy	Anticancer drug synergy	FNN	To predict anticancer drug synergy	CSV / CSV	https://github.com/KristinaPreuer/DeepSynergy	[95]

To investigate these pharmacogenomics associations, statistical, such as the analysis of variance (ANOVA) test, is utilised. This can identify, for example, oncogenic changes that occur in patients, which are indicators of drug-sensitivity variances in cell lines. In order to move beyond the drug’s relations to the actual drug reaction predictions, numerous statistical and machine learning methods can be employed, from linear regression models to nonlinear ones, such as kernel methods, neural networks and SVM. A central weakness of these approaches is the massive number of inputs feature alongside the low sampling, such as in standard gene expression analysis, and the total number of input genes (or features) exceeds the sample number. An up-to-date strategy to overcome the low sampling number issue is to engage multitasking models [96].

Deep learning methods are reportedly well suited to treatment response prediction tasks based on cell-line omics datasets [95, 97]. One of the examples is, DrugCell, a visible neural network (VNN) interpretation model for the structure and function of human cancer cells in therapy response. It pairs the model’s central mechanisms to the human cell-biology structure. Permitting the prediction of any drug response within any cancer then smartly plans the successful combination of treatments. DrugCell was developed to capture both elements of therapy response in an explainable model with two divisions, the VNN-integrating cell genotype and the artificial neural network (ANN)-integrating drug design. The first VNN model inputs comprise text files of the hierarchal association between molecular sub-systems in human cells, which contain 2086 biological process standards in the Gene Ontology (GO) database. The second ANN model inputs were conventional ANN integrating text files of the Morgan fingerprint of medicine, the chemical structure of a canonical vector symbol. The outputs from these two divisions were combined into a single layer of neurons that produced the response of a given genotype to a certain therapy. The prediction accuracy of each drug separately revealed a drug sub-population with significant accuracy. This, in turn, competes with the state-of-the-art regression methods applied in previous models to predict the drug response. Additionally, comparing DrugCell with a parallel neural network model trained merely on drug design and labelled tissue extremely outperformed the tissue-based model. This means that DrugCell has learned data from somatic mutations exceeding the tissue-only method [26]. Another recent model called DeepBL is based on deep learning architecture executed based on Small VGGNet structure (a type of CNNs) and TensorFlow library. This approach detects the beta-lactamases (BLs) and their varieties that provide resistance to beta-lactam antibiotics, with protein sequences as inputs. It is based on well-interpreted massive RefSeq datasets covering > 39 K BLs extracted from the NCBI database. Comparing this model with the other conventional machine learning-based algorithms, including SVM, RF, NB and LR, DeepBL outperformed them after evaluation on an independent test set comprising more than 10 K sequences [98]. Until very recently, deep learning applications in pharmacogenomics remained under consideration.

## Deep learning algorithms/techniques used in genomics

The accomplishment of the recent, attainable models mentioned in deep learning tools/software/pipelines in genomics section suggests that deep learning is a powerful technique in genomic research. Here, we focus on deep learning algorithms recently applied in genomic applications: convolutional neural networks (CNNs), feedforward neural networks (FNN), natural language processing (NLP), recurrent neural networks (RNNs), long short-term memory networks (LSTMs), bidirectional long short-term memory networks (BLSTMs) and gated recurrent unit (GRU; Table 6; Fig. 1).

**Table 6:** Deep learning algorithms in genomics and their original development and applications

ANN Algorithms	Natural Language Processing (NLP)	Feedforward neural network	Convolutional neural network (CNN)	Recurrent neural networks (RNNs)	Bidirectional long short-term memory networks (BLSTMs)	Long short-term memory networks (LSTMs)	Gated recurrent unit (GRU)
Algorithm Inventor	Applied in dictionary look-up system developed at Birkbeck College, London	Frank Rosenblatt	It was named as “neocognitron “ by Fukushima	Rumelhart, Hinton and Williams	Schuster and Paliwal	Hochreiter and Schmidhuber	Cho et al
Year of Development	1948	1958	1980	1986	1997	1997	2014
Year of Initial Genomics’ Function	1996	1993	2015	2005	2015	2015	2017
First User in Genomics	Schuler et al	S Eskiizmililer	Alipanahi et al	Maraziotis, Dragomir and Bezerianos	Quang and Xie	Quang and Xie	Angermueller et al
First Genomic Application	Entrez databases	Karyotyping architecture based on Artificial Neural Networks	DeepBind	Predicting the complicated causative associations between genes from microarray datasets based on recurrent neuro-fuzzy technique	DanQ model	DanQ model	DeepCpG
Genomic Function Exemplar(s)	Genetic counsellors AI-based chatbots and EPIs prediction	Karyotyping, Prenatal diagnostic for early detection of aneuploidy syndrome	﻿Prediction of variant impacts on expression and disease risk, predicting drug response of tumours from genomic profiles, and pharmacogenomics	Predicting transcription factor binding sites, for Alignment and SNV identification	DNA function predictions and prediction of protein localisation, predict miRNA precursor	Enhancer–promoter interaction (EPI) prediction	Enhancers and methylation states predictions
Landmark References	[128, 169, 170]	[171–173]	[97, 111, 174–176]	[24, 116, 118, 177, 178]	[122, 123, 179, 180]	[16, 121, 123]	[126, 181]

Deep learning is a contemporary and rapidly expanding subarea of machine learning. It endeavours to model concepts from wide-ranging data by occupying multi-layered DNNs, hence creating data logic, such as pictures, sounds and texts. Generally, deep learning has two features: first, the structure of nonlinear processing parts is multiple layers, and second, the feature extraction fashion on each layer is either the supervised or unsupervised method [99]. In the 1980s, the initial deep learning architecture was constructed on artificial neural networks (ANNs) [100], but the actual power of deep learning developed outward in 2006 [101, 102]. Since then, deep learning has been functional in various arenas involving genomics, bioinformatics, drug discovery, automated speech detection, image recognition and natural language processing [6, 13, 103].

Artificial neural networks (ANNs) were motivated by the human brain’s neurons and their networks [104]. They consist of clusters of fully connected nodes, or neurons, demonstrating the stimulus circulation of synapses in the brain through the neural networks. This architecture of deep learning networks is utilised for feature extraction, classification, decreased data dimensions or sub-elements of a deeper framework such as CNNs [105].

Multi-omics study generates huge volumes of data, as mentioned earlier, basically because of the evolution that has been pursued in genomics and improvements in biotechnology. Symbolic examples involve the high-throughput technology, which extent thousands of gene expression or non-coding transcription, such as miRNAs. Moreover, the genotyping platforms and NGS techniques and the associated GWAS that generates measurable gene expression reports, such as RNA-Seq, discover numerous genetic variants, together with further genomic modifications in various populations [11]. However, some DL models rely purely on DNA sequence datasets that seemingly lack the power to create predictions of a cell-line-exclusive method due to the identical DNA sequencing of various cell lines. In order to overcome this deficiency, several hybrid deep learning models have been advised and revealed obvious enhancement in certain studies through joining DNA sequencing data with biological experiments information [84].

*Feedforward Neural Networks (FNNs)* Are a type of artificial neural network that consists of one forward direction network starting from input layers, crossing the hidden layers and reaching to the output layer, without forming loops such as RNNs [106]. It is used in genomics to comprehend the expression of target genes from the expression of landmark genes using the D-GEX model [12]. Moreover, active enhancers and promoters have been predicted across the human genome utilising the DECRES model [107]. Moreover, anticancer drug synergy predictions have been made via the DeepSynergy model [95].

*Convolutional Neural Networks (CNNs)* Also called ConvNet, CNN is a deep learning algorithm that has a deep feedforward architecture consisting of various building blocks, such as convolution layers, pooling layers and fully connected layers [97, 108]. It illustrates a fully connected network since each node in a single layer is fully connected to the entire node of the next layer. The convolution units in the CNN layers can obtain the input data from units of the earlier one, which all together generate a prediction. The key principle of such deep construction is that massive processing and connection feature represents inferring nonlinear association between both inputs and outputs [109, 110]. The most common analysis uses of CNNs were applied in graphical images and were initially considered a fully automated image network interpreter for classifying handcraft fonts [105].

For genomic functions, CNNs considered the dominant algorithm utilised genomic information (Fig. 2). The primary CNN implementation, DeepBind, was proposed by [111] for binding protein predictions and showed greater prediction power than conventional models (Table 6). More examples of CNN are used as a single algorithm in gene expression, and regulations include the DeepExpression model, which has been effectively used to predict gene expression using promoter sequences and enhancer–promoter interactions [112]. The SpliceAI model was introduced to identify splice function from pre-mRNA sequencing [71]. Further, the SPOT-RNA model was developed for predicting RNA secondary structure [16]. CNN was also used for DNA sequencing in call genetic variants, such as Clairvoyante, Intelli-NGS and DeepSV models [52, 54, 113]. In epigenomics, the DeepTACT model was used for predicting the 3D chromatin interactions [82], and the Basenji model was employed for predicting cell-type-specific epigenetic and transcriptional profiles in large mammalian genomes [114]. In disease variants, the ExPecto model was used to predict tissue-specific transcriptional effects of mutations/functions [32], and the DeepWAS model was used to identify disease or trait-associated SNPs [19]. Finally, in pharmacogenomics applications, CNN was utilised to create the DrugCell model for drug response and synergy predictions [26]. Additionally, the DeepDrug3D model was obtained for characterising and classifying the 3D protein binding pockets [115].

Additionally, CNN algorithms were combined with other algorithms to build up efficient approaches in epigenomics, combining CNN with GRU to predict the methylation states from single-cell data [83], while in terms of gene expression and regulation, [74] linked CNN algorithms with MLP in the DECRES model to predict active enhancers and promoters across the human genome. Besides, [116] used CNN with RNN algorithms in a DNA sequencing application to create the DAVI model and identify NGS read variants.

*Recurrent neural networks (RNNs)* are ANNs with a recurrent layer consisting of typical recurrent layers that enable state updates of past and current inputs with feedback connections. They are distinguished by the internal cycle connections between recurrent layer units and are concerned with sequential datasets [117, 118]. Recurrent neural networks have regularly expended for the task that comprised in learning sequencing datasets, such as translation languages and recognising speech. However, it has not been utilised widely on DNA sequencing data which is the data style where the order link between bases are crucial for its assessment [119]. Maraziotis et al. [24] initiated RNN implementation in genomics using microarray experimental data based on recurrent the neuro-fuzzy protocol to infer the complicated causative relationship between genes by predicting the time-series of gene expression (Table 6).

Most RNNs are applied in genomics combined with other algorithms, such as CNNs. For example, to identify NGS read variants, the DAVI model introduced the combination of CNN and RNN algorithms [116]. The FactorNet model was designed based on both CNN and RNN algorithms and raised to predict the cell-type-specific transcriptional binding factors (TFBSs) [120]. However, CNN algorithms are perfect at capturing local DNA sequence patterns; contrastingly, RNN derivatives, such as LSTM, are ideal for capturing long-distance dependencies between sequence datasets [119].

*Long short-term memory networks (LSTMs)* are standard recurrent cells with “gates” to handle long-term dependency tasks [118]. They deliberate to prevent long-term dependency difficulties through their competence in acquiring long-term dependencies. It has a node, input gate, output gate and forget gate as core LSTM unit. The node considers values through certain time gaps, whereas the input and output gates control information flow [121]. The preliminary implementations of LSTM algorithms in genomics advised the SPEID model, which used a pattern of deep learning algorithms utilising both LSTM and CNN for EPI predictions (Table 6; [18]). Park et al.[122] obtained DeepMiRGene, a fusion of the RNN and LSTM models, to predict miRNA precursors.

*Bidirectional Long Short-Term Memory Networks (BLSTMs) In* BLSTM, two RNNs with two hidden layers (forward and backward layers) can be trained in both time directions in parallel to enable the previous context usage that cannot be accomplished via standard RNNs [118]. Quang et al. [123] expressed the DanQ model, the original employment in genomics that predicted DNA function directly from sequence data developed from CNN and BLSTM constructions (Table 6). Later, [124] presented DeepCLIP, also utilising CNN and BLSTM, to predict the effect of mutations on protein–RNA binding.

*Gated Recurrent Unit (GRU)* is categorised as a variant of the LSTM algorithm with cell has only “two gates”: the update gate and reset gate [118]. It couples neural networks opposing each other. The first network produces artificial, accurate information, while the second estimates the validity of the information [125]. It was initially applied in gene expression and regulation by [126], who presented the BiRen model, an architecture consisting of RNNs, CNNs and GRUs, to predict enhancers (Table 6). After, the DeepCpG model appeared, combining CNN and GRU frameworks to predict the methylation states from single-cell data [83].

*Natural Language Processing (NLP)* It examines the computers usage to recognise human languages for the purpose of executing beneficial tasks [127]. In the field of NLP, in fact, the “distributed representations” technique is utilised in several state-of-the-art DL models [128]. For example, the word2vec model is an achieved NLP that utilises the distribution representation process, “neural embedding”. This is because of the embedding task that is frequently expressed through neural networks beside numerous parameters. The aim of word embedding is to convey linear mapping and then generate a direct advantage of representing a single word, thereby distinguishing vectors in continuous space and hence become open for backpropagation-based methods in neural networks [129]. In terms of deep learning demands in the field of gene expression and regulation, Du et al. (2019) explored the Gene2vec model, an idea of distributed representation of genes. It engages genes’ natural contexts and their expression and co-expression patterns from GEO data. The essential layer of a multilayer neural network uses the embedded gene, which predicts gene-to-gene interactions with a 0.72 AUC score. This is an interesting outcome because the initial model input is the names of two genes merely. Thus, the distributed representation of genes technique is burdened with rich indications about gene function [130]. Another NLP implementation in the same field was shown by Zeng et al. (2018), who combined NLP with GBRT and introduced the EP2vec model to EPIs.

*Graphical Neural Network (GNN)* Due to the emerging biological network data sets in genomics, graph neural network has been evolved as an important deep learning method to tackle these data sets[131]. GNN was proposed by Gori et al. (2005) as a novel neural network model to tackle graph structure data [132]. Out of many applications of GNN in analysing multi-omics data, the few salient ones are disease gene prediction, drug discovery, drug interaction network, protein–protein interaction network and biomedical imaging. GNN is capable of modelling both the molecular structure data [133] and biological network data[134].

## Deep learning resources for genomics

We collected the most efficient user-friendly genomic resources developed based on deep learning architectures (Table 7). The adoption of various deep learning solutions and models is still limited, despite the enormous success of these tools in genomics and bioinformatics. One reason for this is the lack of deep learning-based published protocols to adapt to new, heterogeneous datasets requiring significant data engineering [135]. In genomics, high-throughput data (e.g. WGS, WES, RNA-seq, ChIP-seq, etc.) are utilised to train neural networks and have become typical for disease predictions or understanding regulatory genomics. Similarly, developing new DL models and testing current models on new datasets face great challenges due to the lack of inclusive, generalisable, practical deep learning libraries for biology [136]. In this respect, software frameworks and genomic packages are necessary to allow rapid progress in adopting a novel research question or hypothesis, combining original data or investigating using different neural network structures [135]. In order to facilitate the DL model implementation in genomics, the following software packages or libraries could become critical for genomic scientists and biomedical researchers.

**Table 7:** Deep learning packages and resources

Resource Name	Category	Application	Date created	Link	Free/paid
*Libraries*					
Janggu^a^	Python package	facilitates deep learning in the context of genomics	2020	https://github.com/BIMSBbioinfo/janggu	Free
ExPecto^a^	Python-based repository	Contains code for predicting expression effects of human genome variants ab initio from sequence	2018	https://github.com/FunctionLab/ExPecto	Free
Selene^a^	PyTorch-based Library	A library for biological sequence data training and model architecture development	2019	https://selene.flatironinstitute.org/	Free
Pysster^a^	TensorFlow-based Library	Used for learning sequence and structure motifs In biological sequences using convolutional neural networks	2018	https://github.com/budach/pysster	Free
Kipoi^a^	Python package	Kipoi is an API and a repository of ready-to-use trained models for genomics	2019	https://github.com/kipoi/kipoi http://kipoi.org/	Free
*Compute platform*					
Google Colaboratory (Colab)	PnP GPUs	Colab allows anybody to write and execute arbitrary python code through the browser, and is especially well suited to machine learning, data analysis and education	2017	https://colab.research.google.com/	Free
IBM Cloud	Cloud service	Cloud computing platform; Design complex neural networks, then experiment at scale to deploy optimised learning models within IBM Watson Studio	2011	https://www.ibm.com/cloud	Free tier Cost tier
Google CloudML	PnP GPUs	For extreme scalability in the long run	2008	https://cloud.google.com/ai-platform	Paid
Vertex AI	AI platform	Google Cloud’s new unified ML platform	2021	https://cloud.google.com/vertex-ai	
Amazon EC2	Cloud service	A website facility which delivers secure, scalable compute power in the cloud	2006	https://aws.amazon.com/ec2/	Free Paid

^a^These deep learning libraries/packages are specific to Genomic application

*Janggu* is a deep learning python library based on deep CNN for genomic implementations. It aims at a data-procuring facility and model assessment by supporting flexible neural network prototype models. The Janggu library provides three use cases: transcriptional factor predictions, utilising and enhancing the published deep learning designs and predicting the CAGE-tag count normalisation of promoters. This library offers easy access and pre-processing to convert data from standard file formats (e.g. FASTA, BAM, Bigwig, BED and narrowPeak) to BigWig files [135].

*Selene* is a deep learning library based on PyTorch for biological sequence data training and model architecture development. Selene supports the prediction of genetic variant effects and visualises the variant scores as a Manhattan plot. It also automatically generates training, testing and validation split from the given input dataset. Further, Selene automatically trains the data and can examine the model on a test set, thereby producing a visualised figure to display the model’s performance [137].

*ExPecto* is a variant prioritisation model for predicting the gene expression levels from a broad regulatory region (~ 40 kb) range of promoter-proximal sequencing regions. It relies on CNN to convert the input sequences into epigenomic features. ExPecto facilitates rare variants or unprecedented variants prediction. This is because of its unique design architecture, which does not utilise any variant information during the training process. ExPecto processes VCF files and outputs CSV files [138].

*Pysster* is a python library package based on CNN for biological sequencing data training and classification. Pysster provides automatic hyperparameter optimisation and motif visualisation options along with their position and class enrichment information [139].

*Kipoi* (Greek for “gardens”; pronounced “kípi”) is a genomic repository for sharing and reusing trained genome-related models. Kipoi provides more than 2 K distinctly trained models from 22 different studies covering significant predictive genomic tasks. The prediction includes chromatin accessibility determination, transcription factor binding and alternative splicing from DNA sequences [136].

Implementation of these deep learning, genome-based libraries/packages requires accessing the computer power and familiarity with web-based resources (Table 7). Several major cloud-computing platforms have proposed on-demand GPU access in user-friendly manners, including Google CloudML, IBM cloud, Vertex AI and Amazon EC2 [140–142]. User configuration and the installation of the appropriate environments for general GPU coding are required in these cloud-based machines. Concurrently, for users who need to avoid semi-manual setup methods, an expert plug-and-play (PnP) platform GPU access is offered, such as Google Colaboratory (Colab). Google Colab is considered the simplest alternative python-based notebook and provides free K80 GPU utilisation for 12 continuous hours [143, 144]. Links to the resources (packages/libraries and web platforms) for the application of deep learning in genomics are provided in Table 7.

## Conclusion

This manuscript catalogues different deep learning tools/software developed in different subareas of genomics to fulfil the predictive tasks of various genomic analyses. We discussed, in detail, the data types in different genomics assays so that readers could have primary knowledge of the basic requirements to develop deep learning-based prediction models using human genomics datasets. In the later part of the manuscript, different deep learning architectures were briefly introduced to genomic scientists in order to help them decide the deep learning network architecture for their specific data types and/or problems. We also briefly discussed the late application of the deep learning technique in genomics and its underlying causes and solutions. Towards the end of the manuscript, various computational resources, software packages or libraries and web-based computational platforms are provided to act as pointers for researchers to create their very first deep learning model utilising genomic datasets. In conclusion, this timely review holds the potential to assist genomic scientists in adopting state-of-the-art deep learning techniques for the exploration of genomic NGS datasets and analyses. This will certainly be beneficial for biomedicine and human genomics researchers.

## Data Availability

Not applicable.

## Abbreviations

NGS: Next-generation sequencing
WGS: Whole genome sequencing
WES: Whole exome sequencing
SMS: Single-molecule sequencing
RNA-seq: RNA sequencing
ChIP-seq: Chromatin immunoprecipitation sequencing
PacBio: Pacific biosciences
ONT: Oxford nanopore technology
MPRAs: Massively parallel reporter assays
miRNA: MicroRNAs
GWAS: Genome-wide association study
PSI: Percent selected index
HGVS: Human genome variation society
IMSGC: International multiple sclerosis genetics consortium
VUS: Variant of uncertain significance
CADD: Combined annotation dependent depletion
GATK: Genomic Analysis ToolKit
BAM: Binary alignment map
VCF: Variant call format
FASTA: Text-based format for either nucleotide sequences or amino acids
BED: Browser extensible data
CSV: Comma-separated values
CAGE: Cap analysis of gene expression
GEO: Gene expression omnibus
EPI: Enhancer–promoter interaction
TFBS: Transcription factor binding sites
DL: Deep learning
ML: Machine learning
DNN: Deep neural network
MLP: Multilayer perceptron
CNN: Convolutional neural networks
RNN: Recurrent neural network
LSTM: Long short-term memory network
BLSTM: Bidirectional long short-term memory network
ANN: Artificial neural network
FNN: Feedforward neural networks
NLP: Natural language processing
GRU: Gated recurrent unit
VGGNet: Visual geometry group networks
GBRT: Gradient boosted regression trees
LR: Linear regression
RF: Random forest
NB: Naive Bayes
DBN: Deep belief networks
SVR: Support vector regression
AUC: Area under the curve
auPR: Area under the precision–recall curve
auROC: Area under the receiver operating characteristic
